# 15-Hydroxyprostaglandin Dehydrogenase Is a Predictor of Stroke-Associated Pneumonia

**DOI:** 10.3389/fneur.2022.893624

**Published:** 2022-05-26

**Authors:** Yunfei Xu, Haoduo Qiao, Shun Yang, Lin Zhou, Yao Zhao, Qing Xu, Shuying Miao, Dun Yuan, Jie Zhao, Ying Liu

**Affiliations:** ^1^Department of Pathophysiology, Xiangya School of Medicine, Central South University, Changsha, China; ^2^Department of Neurosurgery, Xiangya Hospital, Central South University, Changsha, China; ^3^Sepsis Translational Medicine Key Lab of Hunan Province, Central South University, Changsha, China; ^4^China-Africa Research Center of Infectious Diseases, Central South University, Changsha, China; ^5^Department of Pathology, Nanjing Drum Tower Hospital, Nanjing University Medical School, Nanjing, China

**Keywords:** 15-PGDH, stroke, pneumonia, PGE2, predictor

## Abstract

**Background and Purpose:**

Stroke is a serious fatal and disabling disease. Stroke-associated pneumonia (SAP) is the most common complication of stroke, which may further aggravate the stroke. The prevention and early prediction of SAP is a key clinical strategy. 15-hydroxyprostaglandin dehydrogenase (15-PGDH) is involved in pneumonia, while its relationship with SAP has yet to be determined. Therefore, we investigated the predictive value of 15-PGDH for SAP and visualized their relationship.

**Methods:**

Stroke patients were recruited and divided into SAP group and Non-SAP group. Baseline demographic and clinical data were obtained from the medical record system, blood samples were collected to detect relevant variables and 15-PGDH levels. Patient characteristics were compared with a t-test. Binary logistic regression analysis was performed to determine the predictive value of 15-PGDH for SAP. Restricted cubic splines (RCS) were performed to visualize the relationship between 15-PGDH and SAP risk. Finally, the SAP patient characteristics between the severe group and mild group were compared.

**Results:**

50 patients were enrolled and divided into SAP group (*n* = 26) and Non-SAP group (*n* = 24). 15-PGDH in the SAP group was lower than that in the Non-SAP group (0.258 ± 0.275 vs. 0.784 ± 0.615, *p* = 0.025). Binary logistic regression analysis revealed that the lower 15-PGDH, the higher the risk of SAP (OR = 0.04, 95%CI, 0.010–0.157, *p* < 0.001). The RCS model showed the L-shaped relationship between 15-PGDH and SAP.

**Conclusions:**

In stroke patients, serum 15-PGDH is a valuable biomarker for predicting SAP. There is an L-shaped relationship between the level of 15-PGDH and the risk of SAP.

## Introduction

Stroke is a common acute cerebrovascular disease, which is the second leading cause of death and the third-leading cause of death and disability combined globally (1). The high mortality rate of stroke is largely attributable to its complications. Stroke-associated pneumonia (SAP) is the most common complication of stroke, occurring most often within the first week in 8.6% to 14.3% of stroke patients (2–4). Stroke activates inflammatory cascades in the brain, such as the excessive release of cytokines, then stimulates the central immune system to produce anti-inflammatory signals. Subsequently, the efferent vagus nerve is activated and inhibits cytokine production, ultimately leading to the suppression of systemic immunity. Stroke-induced immunosuppression increases the susceptibility of stroke patients to infection, accompanied by decreased pulmonary clearance and impaired secretion drainage caused by the alteration in tracheal endothelia after stroke, ultimately leading to the occurrence of SAP (5–7). At the same time, risk factors such as stroke severity, advanced age, hypertension, and smoking history also contribute to the occurrence of SAP (8, 9). SAP further increases the severity of the stroke, lengthens hospital stay, increases financial burden, worsens the functional outcome, and increases patient mortality (9, 10). Prevention and early diagnosis or prediction of SAP is an important clinical strategy. However, current methods are less specific and sensitive, making the early diagnosis of SAP become a big difficulty (3, 11). There is still great value in exploring the early markers of SAP.

Prostaglandin E2 (PGE2) is a key mediator of inflammation, its elevation is a response to inflammation (12). 15-hydroxyprostaglandin dehydrogenase (15-PGDH) is the sole degradation enzyme of PGE2 by oxidizing it to inactive 15-keto PGE2 (13). The decrease of 15-PGDH can lead to an increase in PGE2, which has been observed in stroke (14). Meanwhile, 15-PGDH is generally identified as a tumor suppressor, such as lung cancer, breast cancer, colorectal cancer, etc (15–17). In addition, 15-PGDH is also involved in lipopolysaccharide-induced acute kidney injury, pulmonary fibrosis, tissue repair, and other pathological processes (18–20). Interestingly, a previous study reported that in pulmonary infection caused by pseudomonas aeruginosa, inhibition of PGE2 overproduction shows a protective effect, in part by raising the level of 15-PGDH (21). However, no report has revealed the level of 15-PGDH in SAP and the relationship between 15-PGDH and SAP. Therefore, we will determine the relationship between 15-PGDH and SAP, and determine whether it can be used as an early marker to predict SAP and provide a basis for early diagnosis of SAP.

## Methods

### Patients Selection

This was a retrospective study, all the patients with hemorrhagic stroke or ischemic stroke were recruited from the Department of Neurosurgery, Xiangya Hospital, Central South University between February 2019 to June 2021. This research was approved by the ethics committee of the Xiangya Hospital of Central South University and the human ethics identification number is 2021101119. All participants involved in this study provided written informed consent.

The inclusion criteria of stroke patients were as follows: (1) age≥18 years; (2) stroke onset within 48 h; (3) acute stroke confirmed by brain magnetic resonance imaging (MRI) or brain computerized tomography (CT). The exclusion criteria were as follows: (1) active infection or pneumonia before admission; (2) patients treated with nonsteroidal anti-inflammatory drugs; (3) patients with incomplete clinical records. Besides, the diagnosis of SAP was performed by two experienced neurologists based on the modified Centers for Disease Control and Prevention criteria of hospital- acquired pneumonia (22), assisted with clinical and laboratory parameters of respiratory infection and was confirmed by chest X-ray or CT (23).

**Figure 1:**
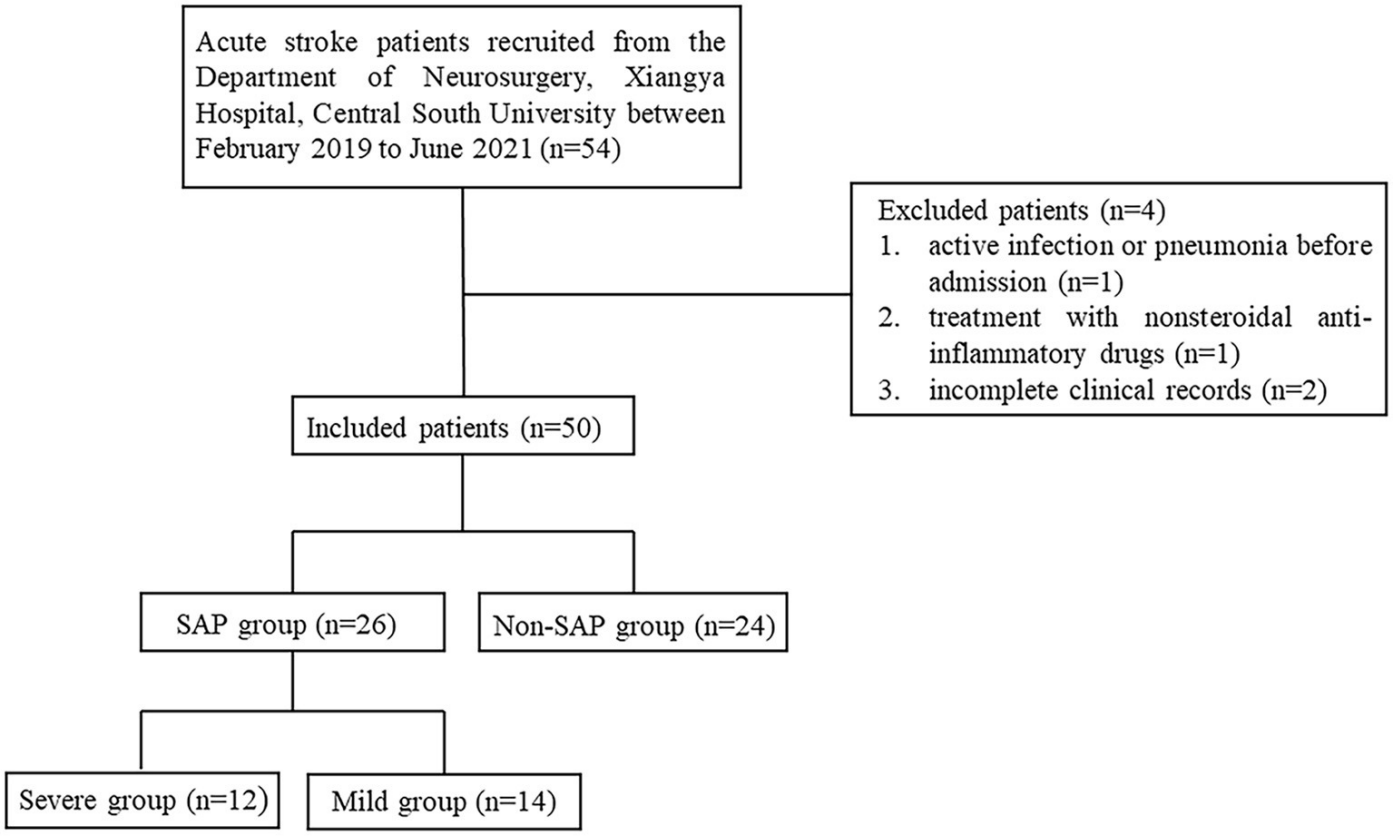
Flowchart of case recruitment. SAP, stroke-associated pneumonia; PSI, pneumonia severity index.

54 Patients Were Recruited, 4 Patients Were Excluded, and 50 Patients Were Included in This Study. Further, 26 Patients Were Diagnosed in the SAP Group and 24 Patients Were Diagnosed in the Non-SAP Group (Figure 1). The SAP Group Was Evaluated by Pneumonia Severity Index (PSI) for Severity and Was Divided Into Severe Group (PSI>90) and the Mild Group (PSI ≤ 90) (24).

### Clinical Characteristics and Laboratory Data Collection

Baseline demographic and clinical data were obtained from the medical record system, mainly including age, sex, risk factors or past medical history such as history of smoking and drinking, hypertension, hyperlipidemia, coronary heart disease, chronic obstructive pulmonary disease (COPD), diabetes mellitus, and nephropathy, as well as ICU admission, hospitalization time, stroke subtype. Glasgow coma score (GCS) was used to assess the severity of stroke at admission. Blood samples were collected the morning after admission and tested for the counts of white blood cell (WBC), red blood cell (RBC), hemoglobin, platelets, neutrophils, and lymphocytes. Further, after SAP diagnosis, PSI was used to assess the severity of SAP.

### Measurement of 15-PGDH

Blood samples were collected the morning after admission. After centrifugation at 2000 g for 10 min, serum was isolated and frozen at −80°C for later measurement. A Human 15-PGDH/HPGD enzyme-linked immunosorbent assay (ELISA) kit (Cat No. ELH-15PGDH-1, RayBiotech) was used to detect the levels of serum 15-PGDH. Detect and calculate the results according to the instructions of the kit.

### Statistical Analysis

All statistical analyses were performed using Statistical Package for the Social Sciences version 26 (SPSS) and R (version 4.1.2). Normally distributed continuous variables were presented as the mean ± standard deviation, while other continuous variables were presented as the median and interquartile range (IQR). Continuous variables were analyzed by *t*-test or the Mann–Whitney U-test. Classified data were presented as percentages and analyzed by the chi-square test or Fisher exact test. Binary Logistic regression analysis was used to confirm the contribution of risk factors to SAP occurrence. The receiver operating characteristic (ROC) curve was used to determine the discrimination ability of 15-PGDH levels to predict SAP. The area under the curve (AUC) was determined from the ROC curve analysis. Restricted cubic splines (RCS) with four knots were performed to visualize the relationship between 15-PGDH and SAP risk. All statistical analyses were two-sided and *p* < 0.05 was set to be a statistical difference.

## Results

### Patient Characteristics

From February 2019 to June 2021, 54 stroke patients were recruited from the Department of Neurosurgery, Xiangya Hospital, Central South University. While 4 patients were excluded, 1 patient due to active infection or pneumonia before admission, 1 patient due to treatment with non-steroidal anti-inflammatory drugs, and 2 patients due to incomplete clinical records. Finally, 50 patients were included in the study. Twenty Six patients were assigned to the SAP group (52%) and 24 to the Non-SAP group (48%). Further, according to PSI, the SAP group was further divided into severe group (*n* = 12) and mild group (*n* = 14). These results were summarized in Figure 1.

As shown in Table 1, age (*p* = 0.011), smoking (*p* = 0.037), the counts of WBC (*p* = 0.034) and neutrophils (*p* = 0.012) and hospitalization time (*p* = 0.039) in SAP group were significantly higher than those in Non-SAP group, and GCS score (*p* = 0.029), the counts of thrombocytes (*p* = 0.032) and lymphocytes (*p* = 0.021) in SAP group were significantly lower than those in Non-SAP group. While other variables including sex, drinking, hypertension, hyperlipidemia, coronary heart disease, COPD, diabetes mellitus, nephropathy, stroke type, the counts of RBC and hemoglobin were not statistically different. Notably, the level of 15-PGDH (*p* = 0.025) in the SAP group was significantly lower than that in the Non-SAP group, indicating that there may be some relationship between 15-PGDH and SAP, which is what we will analyze next.

**Table 1:** Patient characteristics with SAP and Non-SAP with stroke.

**Variables**	**SAP (*n* = 26)**	**Non-SAP (*n* = 24)**	***p*** **-value**
Age, y, mean (SD)	59.2 (11.2)	55 (11.2)	0.011
Sex, male (%)	14 (53.8)	10 (41.7)	0.389
Smoking (%)	10 (38.5)	3 (12.5)	0.037
Drinking (%)	6 (23.1)	2 (8.3)	0.155
Hypertension (%)	15 (57.7)	13 (54.2)	0.802
Hyperlipidemia (%)	2 (7.7)	2 (8.3)	0.934
Coronary heart disease (%)	4 (15.4)	1 (4.2)	0.187
COPD (%)	2 (7.7)	2 (8.3)	0.934
Diabetes mellitus (%)	2 (7.7)	3 (12.5)	0.571
Nephropathy (%)	7 (26.9)	3 (12.5)	0.203
Hemorrhagic stroke (%)	18 (69.2)	14 (58.3)	0.423
GCS, median (IQR)	7.5 (6.3)	11.0 (4.0)	0.029
WBC, 10^9^/L	11.7 (6.4)	8.3 (2.3)	0.034
RBC, 10^9^/L	3.67 (0.73)	3.76 (0.87)	0.676
Hemoglobin, g/L	117.8 (30.2)	115.0 (28.8)	0.739
Thrombocytes, 10^9^/L	176.2 (63.3)	229.7 (104.7)	0.032
Lymphocytes, 10^9^/L	1.03 (0.53)	1.26 (0.77)	0.021
Neutrophil, 10^9^/L	8.90 (6.30)	6.93 (3.38)	0.012
ICU admission (%)	24 (92.3)	19 (79.2)	0.181
hospitalization time, d, median (IQR)	17.00 (18.00)	12.00 (9.25)	0.039
15-PGDH, ng/mL	0.258 (0.275)	0.784 (0.615)	0.025

*SAP, stroke-associated pneumonia; SD, standard deviation; COPD, chronic obstructive pulmonary disease; GCS, Glasgow coma scale; IQR, interquartile range; WBC, white blood cell; RBC, red blood cell; ICU, intensive care unit; 15-PGDH, 15-hydroxyprostaglandin dehydrogenase*.

### 15-PGDH Is Valuable to Predict SAP

A binary logistic regression analysis was performed to determine which variables could be potential for predicting SAP. As shown in Table 2, higher age (OR = 1.057, 95%CI = 1.026–1.090, *p* < 0.001), smoking (OR = 4.375, 95%CI = 2.214–9.010, *p* < 0.001), WBC (OR = 1.283, 95%CI = 1.128–1.459, *p* < 0.001), neutrophil (OR = 1.088, 95%CI = 1.015–1.166, *p* = 0.017) and lower GCS (OR = 0.754, 95%CI = 0.658–0.864, *p* < 0.001), thrombocytes (OR = 0.992, 95%CI = 0.988–0.996, *p* < 0.001), lymphocytes (OR = 0.575, 95%CI = 0.354–0.933, *p* = 0.025) were risk factors of SAP.

**Table 2:** Factors contributed to SAP by the multivariate analysis.

**Variables**	**OR**	**95%CI**	***p*** **-value**
Age	1.057	1.026–1.090	<0.001
Smoking	4.375	2.124–9.010	<0.001
GCS	0.754	0.658–0.864	<0.001
WBC	1.283	1.128–1.459	<0.001
Thrombocytes	0.992	0.988–0.996	<0.001
15-PGDH	0.04	0.010–0.157	<0.001
Neutrophil	1.088	1.015–1.166	0.017
Lymphocytes	0.575	0.354-−0.933	0.025

*SAP, stroke-associated pneumonia; GCS, Glasgow coma scale; WBC, white blood cell; 15-PGDH, 15-hydroxyprostaglandin dehydrogenase; OR, odds ratio; 95%CI, 95% confidence interval*.

**Figure 2:**
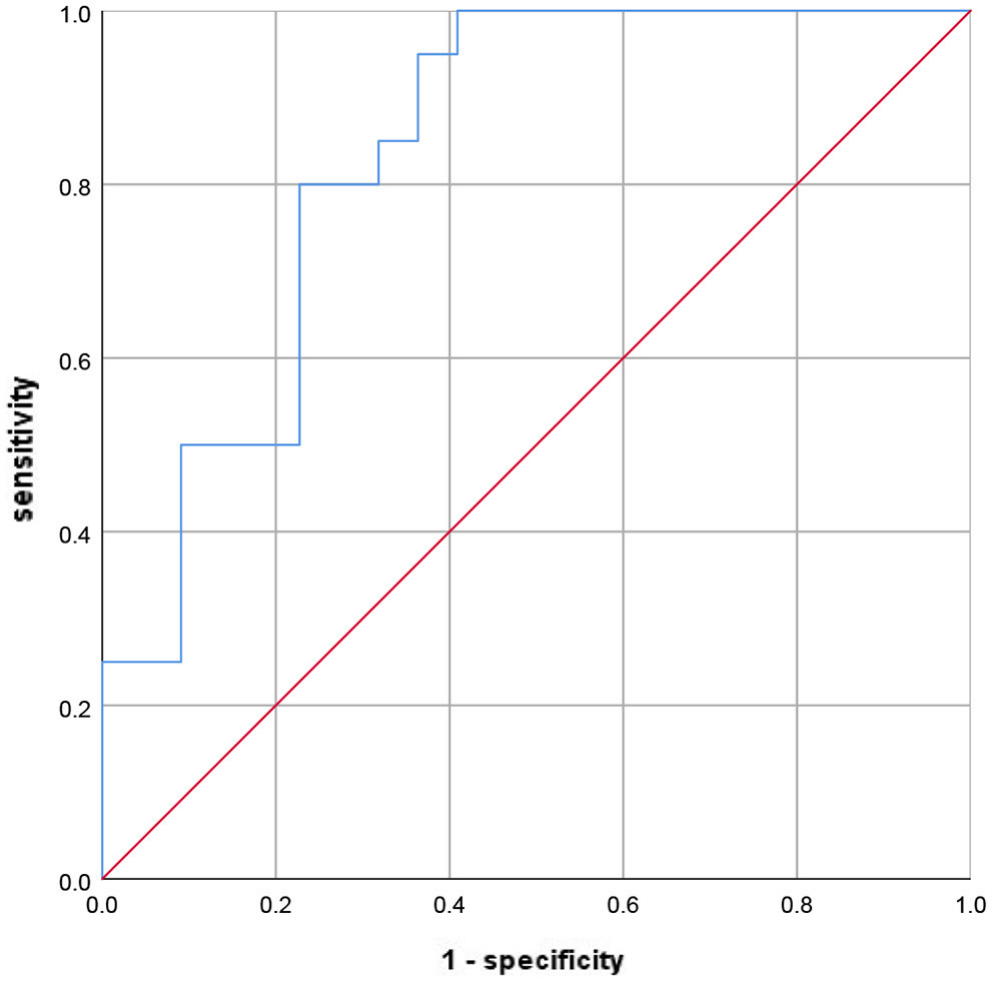
The ROC curve to evaluate the diagnostic value of 15-PGDH for SAP. ROC, receiver operating characteristic; SAP, stroke-associated pneumonia; 15-PGDH, 15-hydroxyprostaglandin dehydrogenase.

Notably, lower 15-PGDH (OR = 0.040, 95%CI = 0.010–0.157, *p* < 0.001) was also a risk factor for SAP. Further, according to the ROC curve in Figure 2, the AUC of 15-PGDH for predicting SAP was 0.836, 95%CI = 0.751–0.922, *p* < 0.001. Besides, the optimal cutoff value of 15-PGDH for SAP was 0.186 ng/mL, with a specificity of 100% and a sensitivity of 59.1%.

**Figure 3:**
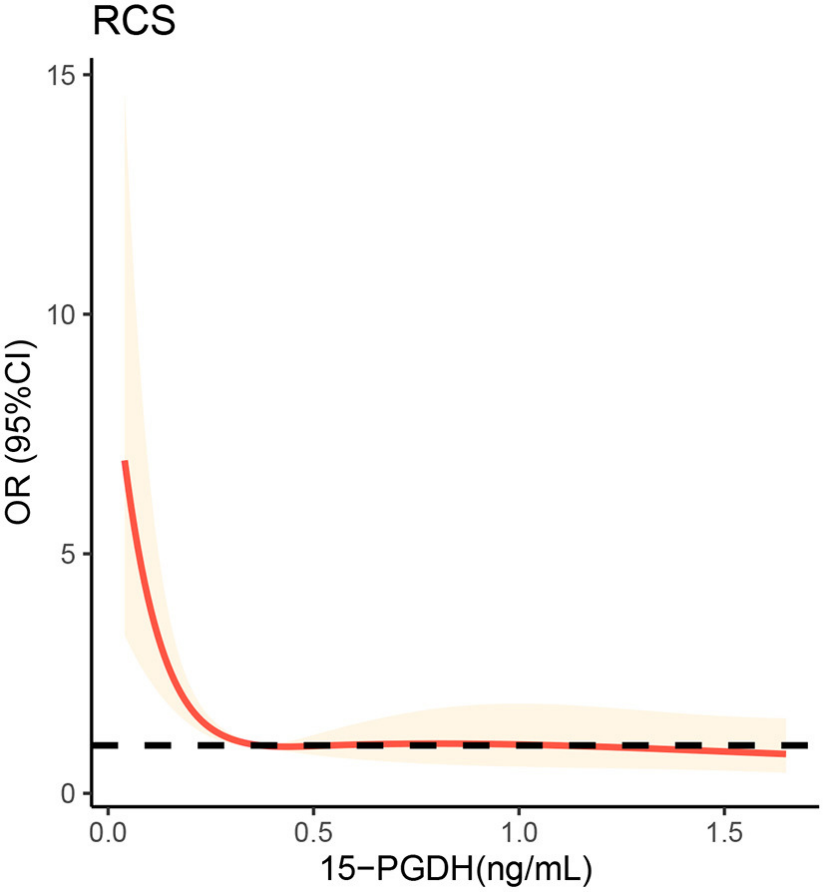
Restricted cubic spline analysis to evaluate the association between serum 15-PGDH levels and risk of SAP. SAP, stroke-associated pneumonia; OR, odds ratio; 95%CI, 95% confidence interval; 15-PGDH, 15-hydroxyprostaglandin dehydrogenase.

To further visualize the relationship between 15-PGDH and SAP risk, restricted cubic spline was next performed. As shown in the Figure 3, the lowest level of 15-PGDH represented the highest risk of SAP. With the increase of 15-PGDH, the risk of SAP decreases continuously. In particular, 0.37 ng/ mL was a point of interest. When it was lower than 0.37 ng/ mL, the risk of SAP increased sharply with the decrease of 15-PGDH, while when it was higher than 0.37 ng/ mL, the risk of SAP increased relatively gentle.

### Patient Characteristics With Severe SAP and Mild SAP

According to PSI, the SAP group was further divided into severe group (*n* = 12) and mild group (*n* = 14). There were some differences between the two groups in terms of patient characteristics in Table 3. Patients in the severe group observed higher age (*p* < 0.001), WBC count (*p* = 0.017) and neutrophils count (*p* = 0.018) than those in the mild group.

**Table 3:** Patient characteristics with severe SAP and mild SAP.

**Variables**	**Severe group**	**Mild group**	***p*** **-value**
	**(*n* = 12)**	**(*n* = 14)**	
Age, y, mean (SD)	64.9 (9.7)	53.5 (9.8)	<0.001
Sex, male (%)	7 (58.3)	7 (50.0)	0.671
Smoking (%)	5 (41.7)	5 (35.7)	0.756
Drinking (%)	3 (25.0)	3 (21.4)	0.829
Hypertension (%)	8 (66.7)	6 (42.9)	0.225
Nephropathy (%)	4 (33.3)	3 (21.4)	0.495
GCS, median (IQR)	7.5 (3.5)	9.0 (3.0)	0.376
WBC, 10^9^/L	12.2 (7.9)	9.3 (4.2)	0.017
Thrombocytes, 10^9^/L	155.9 (47)	193.5 (68.8)	0.002
Lymphocytes, 10^9^/L	0.98 (0.53)	1.06 (0.34)	0.391
Neutrophil, 10^9^/L	10.4 (8.2)	7.5 (4.1)	0.018
Hospitalization time, d, median (IQR)	21 (24.5)	20 (12.5)	0.786

*SAP, stroke-associated pneumonia; SD, standard deviation; GCS, Glasgow coma scale; IQR, interquartile range; WBC, white blood cell*.

## Discussion

Two important findings are presented in this study. First, lower serum 15-PGDH increases the risk of SAP in stroke patients. Second, there is an L-shaped relationship between 15-PGDH and SAP risk. Once 15-PGDH is lower than the critical value, SAP risk will be significantly increased.

Stroke is an acute neurovascular disease with high morbidity, mortality and disability. SAP is the most common complication of stroke, usually manifests within 7 days after the onset of stroke (3). The complication of SAP further aggravates the severity of stroke, prolonged hospitalization, worsen the prognosis, and increase mortality (6, 10). The occurrence of SAP increases the difficulty of stroke treatment. Therefore, the prevention and early prediction or diagnosis of SAP should not be ignored in the treatment of stroke patients. Our study demonstrates that advanced age, smGoking, higher WBC and neutrophils, and lower GCS, thrombocytes, and lymphocytes are all risk factors for SAP. In addition, previous studies have found that dysphagia (25), aspiration (26), invasive treatments, antacids, hypertension, diabetes, pulmonary diseases and so on are also risk factors for SAP (27). Prevention and early diagnosis of so many risk factors are an important part of stroke and SAP management.

Inflammation-related indicators are the most important predictive markers of SAP and contribute greatly to the early diagnosis of SAP. Monocyte to lymphocyte ratio (28), neutrophil to lymphocyte ratio (24) and platelet to lymphocyte ratio (29) have been proven to be valuable predictors of SAP markers. PGE2 is an important inflammatory mediator involved in many inflammatory processes. As the degradation enzyme of PGE2, 15-PGDH is potential to become one predictive marker of SAP. In our study, serum 15-PGDH level in patients with SAP were significantly lower than those without SAP. Binary logistic regression analysis proved that low 15-PGDH was a risk factor for SAP. With an AUC of 0.836, 15-PGDH is undoubtedly a valuable predictor of SAP. Furthermore, RCS visualizes the relationship between 15-PGDH and SAP risk as L-shaped, which may contribute significantly to the prediction and treatment of SAP. It should be noted that the 15-PGDH concentration of 0.37 ng/mL is a critical threshold. When 15-PGDH is lower than 0.37 ng/mL, its decrease will sharply increase the risk of SAP, while when higher than 0.37 ng/mL, this trend is very gentle. Therefore, when assessing the risk of SAP, on the premise of avoiding the reduction of 15-PGDH, it is more important to ensure that 15-PGDH should not be lower than 0.37 ng/mL, which may be critical for SAP prevention.

Admittedly, there are some limitations to our research. First, since our study is a retrospective study, it is difficult to draw a causal relationship between 15-PGDH and SAP, which requires further verification by subsequent studies. Second, we did not collect enough patients, which may lead to partial results bias. For example, the incidence of SAP was higher than previously reported. Third, although we compared the characteristics of SAP patients in the severe group and mild group, it is unclear whether the level of 15-PGDH is predictive of the severity of SAP. Furthermore, we did not analyze patients with different stroke types separately, and there may be some different findings.

## Conclusion

In stroke patients, serum 15-PGDH is a valuable biomarker for predicting SAP. There is an L-shaped relationship between the level of 15-PGDH and the risk of SAP. Once lower than 0.37 ng/mL, the risk of SAP will increase rapidly.

## Data Availability Statement

The original contributions presented in the study are included in the article/supplementary material, further inquiries can be directed to the corresponding authors.

## Ethics Statement

The studies involving human participants were reviewed and approved by the Ethics Committee of the Xiangya Hospital of Central South University. The patients/participants provided their written informed consent to participate in this study.

## Author Contributions

DY, YL, and JZ designed and directed the project. SY collected clinical samples. HQ collected baseline demographic and clinical data. YX, LZ, YZ, and QX performed the experiments. YX wrote the manuscript.

## Funding

This work was supported by the National Natural Science Foundation of China (Nos. 82172147, 81571880, 81373147, 30901555, 30972870, and 81360080), Natural Science Foundation of Hunan Province (2021JJ30900 and 2016JJ2157), and the Fundamental Research Funds for the Central Universities of Central South University (2020zzts222). All funding bodies were involved in the study design, collection, analysis, interpretation of data, and writing the manuscript.

## Conflict of Interest

The authors declare that the research was conducted in the absence of any commercial or financial relationships that could be construed as a potential conflict of interest.

## Publisher's Note

All claims expressed in this article are solely those of the authors and do not necessarily represent those of their affiliated organizations, or those of the publisher, the editors and the reviewers. Any product that may be evaluated in this article, or claim that may be made by its manufacturer, is not guaranteed or endorsed by the publisher.

## Abbreviations

15-PGDH: 15-hydroxyprostaglandin dehydrogenase
AUC: area under the curve
COPD: chronic obstructive pulmonary disease
CI: confidence interval
CT: computerized tomography
ELISA: enzyme-linked immunosorbent assay
GCS: Glasgow coma score
IQR: interquartile range
MRI: magnetic resonance imaging
OR: odds ratio
PGE2: prostaglandin E2
PSI: pneumonia severity index
RBC: red blood cell
RCS: restricted cubic splines
ROC: receiver operating characteristic
SAP: stroke-associated pneumonia
WBC: white blood cell.
